# Copper Oxidation-Induced Nanoscale Deformation of Electromechanical, Laminate Polymer/Graphene Thin Films during Thermal Annealing: Implications for Flexible, Transparent, and Conductive Electrodes

**DOI:** 10.1021/acsanm.4c06372

**Published:** 2024-12-12

**Authors:** Zacary L. Croft, Oscar Valenzuela, Connor Thompson, Brendan Whitfield, Garrett Betzko, Guoliang Liu

**Affiliations:** † Department of Chemistry, Virginia Tech, Blacksburg, Virginia 24061, United States; ‡ Division of Nanoscience, Academy of Integrated Science, Virginia Tech, Blacksburg, Virginia 24061, United States; § Macromolecules Innovation Institute, Virginia Tech, Blacksburg, Virginia 24061, United States; ∥ Department of Chemical Engineering, Department of Materials Science and Engineering, Virginia Tech, Blacksburg, Virginia 24061, United States

**Keywords:** polymer/graphene, thin films, thermal annealing, oxidation, deformation, mechanics

## Abstract

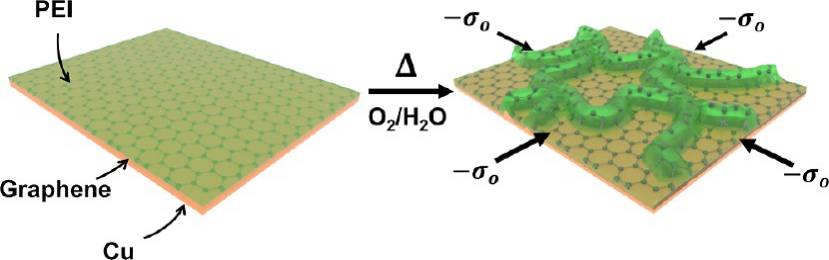

The transfer of large-area, continuous, chemical vapor deposition (CVD)-grown graphene without introducing defects remains a challenge for fabricating graphene-based electronics. Polymer thin films are commonly used as supports for transferring graphene, but they typically require thermal annealing before transfer. However, little work has been done to thoroughly investigate how thermal annealing affects the polymer/graphene thin film when directly annealed on the growth substrate. In this work, we demonstrate that under improper annealing conditions, thermal annealing of poly(ether imide)/single-layer graphene (PEI/SLG) thin films on Cu causes detrimental nanoscale structural deformations, which permanently degrade the mechanical properties. Furthermore, we elucidate the mechanisms of PEI/SLG deformation during thermal annealing and find that permanent deformations and cracking are caused by Cu substrate oxidation. This study provides an understanding of annealing-induced deformation in polymer/graphene thin films. We anticipate that this knowledge will be useful for further developing defect-free, graphene-based thin film electronics.

## Introduction

Continuous graphene film grown by chemical vapor deposition (CVD) has tremendous potential for flexible, transparent, and conductive electrodes in next-generation electronics due to its atomic thickness, high charge carrier mobility, and tunable bandgap.^1−3^ In micro/nanoelectromechanical systems (MEMS/NEMS), graphene has enabled new acoustic and sensory devices with superior performances owing to its high in-plane strength, electrical conductivity, and out-of-plane flexibility.^4−7^ However, the micro- and nanostructure of graphene (e.g., rippling, wrinkling, and crumpling) determines its properties,^8,9^ and thus, poor control over the graphene microstructure during device fabrication leads to unreliable performance in practical applications. Therefore, it is of critical importance to understand and control the factors affecting the graphene microstructure throughout the manufacturing processes.

Single-layer graphene (SLG), grown on Cu by CVD, is susceptible to micro- and nanoscale structural deformation and is usually not used as a standalone material. Instead, SLG applications require a substrate, such as silicon and polymers. In particular, polymers and polymer-supported adhesives are regularly employed in transferring SLG from the Cu substrate due to their versatility and compatibility in a wide range of processes.^10−12^ However, the transfer of SLG from Cu often alters its local microstructure and influences the performance of SLG.^13−15^ The use of a sacrificial polymer layer as an intermediate support can cause contamination and undesirable electronic doping due to residual polymer chains.^12,13,16^ Additionally, structural defects, such as cracks, folds, and wrinkles, are commonly introduced due to imperfect interfacial adhesion between SLG and the substrate.^17,18^

While various methods have been developed to realize the “clean” transfer of CVD graphene,^13,18−21^ the use of a polymer transfer support directly as the target substrate has several advantages. Unlike using sacrificial supports, the direct-support approach does not require intermediate steps for polymer dissolution/removal.^22^ Consequently, chemical contamination of SLG by residual polymers is minimized, and the complexity of the transfer process is reduced. Additionally, the deposition of polymer supports using a solution-based method, like spin-coating or solution casting, ensures complete interfacial contact between SLG and the substrate. As a result, material properties arising from the interfacial interaction are improved, and transfer-related structural defects (e.g., wrinkles, folds, and cracks) are greatly reduced compared to that in the sacrificial-support approach.

Besides the many methods to prepare polymer thin films such as dip-coating and hot-pressing,^23,24^ one of the most common solution deposition techniques is spin-coating, which is known to impart polymer thin films with residual stresses that are usually unknown and often difficult to measure.^25−27^ Despite the popularity of spin-coating, the impact of deposition-derived residual stress on the long-term reliability and performance of polymer/graphene heterostructures remains unknown. We hypothesize that residual stresses from spin-coating may influence polymer/graphene thin films and affect the thermomechanical properties. Such residual stress would be especially problematic in cases where the polymer support is used directly as the target substrate because any abnormalities in the structure of the polymer support can threaten the integrity of the entire heterostructure. Thus, poor control over the evolution of internal stress during thermal annealing may cause unpredictable microstructural deformations, which may degrade the final properties and performance.

Like many sacrificial-support approaches, the direct-support approach often involves implementing a thermal annealing step before transfer to remove residual solvent and internal stresses.^28,29^ The effect of thermal annealing on the crystalline graphene on Cu substrates has been studied, revealing damage to the graphene crystalline structure due to volume expansion of the oxidized Cu substrate.^30^ However, few have investigated how on-substrate deposition and thermal processing influence the microstructure and properties of laminate polymer/graphene composites. Therefore, in this study, we investigate the influence of thermal annealing on the morphological and mechanical properties of poly(ether imide) (PEI)/SLG laminate thin films prepared via spin-coating deposition on Cu. We show that thermal annealing negatively impacts the mechanical reinforcement between PEI and SLG, depending on the annealing temperature and atmospheric composition. Specifically, when annealed above the glass transition temperature (*T*_g_) of PEI, the film experiences widespread microstructural deformation that negatively impacts the mechanical strength of the composite. Moreover, we show that deformation requires an oxidant-rich atmosphere (e.g., H_2_O and O_2_) and is mediated by the thermomechanical relaxation of the polymer. Therefore, this report calls for caution when selecting the conditions of polymer/SLG film annealing to ensure the integrity and reliability of polymer/SLG laminates for downstream uses.

## Experimental Section

### Fabrication of Poly(ether imide)/Single-Layer Graphene Thin Films

Bilayer thin films of poly(ether imide) and single-layer graphene (PEI/SLG) were created by spin-coating PEI solution over CVD-grown SLG on Cu foil (SLG/Cu, Grolltex) using a spin-coater (WS-650-23B, Laurell). The spin-coating process was achieved in several steps. First, PEI solutions were prepared at a concentration of 50 mg/mL by dissolving PEI pellets (Sigma-Aldrich) in CHCl_3_ (Sigma-Aldrich) on a hot plate at 40 °C under light stirring. Once the PEI was fully dissolved and no solid particles were visible, the PEI solution was used for spin-coating. A ∼4 × 4 cm^2^ sheet of SLG/Cu was adhered to a 2″ diameter silicon wafer along its edges. Then, a gentle flow of compressed dry air was passed over the sample to dislodge and clear any particles loosely adhered to the SLG/Cu surface. The sample was placed in the spin-coater, and the chamber was purged with high-purity N_2_ (Linde Gas, 99.999%) for 1 min. The PEI solution was dispensed over the SLG/Cu surface before spinning, and the foil was spun at 3000 rpm for 60 s under N_2_ to form a thin layer of PEI over SLG/Cu. The as-spun sample was allowed to dry under ambient conditions (e.g., 19–21 °C, 40–60% RH) for at least 3–4 days before any further processing or analysis.

### Characterization of Thin Films

The surface topography and oxidation characteristics of SLG/Cu and PEI/SLG/Cu were evaluated using scanning electron microscopy (SEM) (Gemini LEO 1550, Carl Zeiss) and atomic force microscopy (DriveAFM, Nanosurf and Jupiter XR, Olympus). Atomic force microscopy was performed in AC tapping mode with supersharp silicon probe tips (SSS-NCH, 250–390 kHz, 42 N/m, 125 × 30 × 4 μm^3^) using drive voltages of 600–800 mV, scanning frequencies of 0.4–0.8 Hz, 10–100 gain, and 512 × 512 resolution. Elemental analysis was conducted under SEM (JEOL JSM-IT500HR) with energy dispersive spectroscopy (EDS) detection (Oxford Instruments, AztecLive Automated Microanalysis System). The vibrational spectrum and strain state of SLG were analyzed by Raman spectroscopy (Alpha 300, WiTec) performed using 532 nm laser excitation. Evaluation of microstructural deformation in PEI/SLG was performed by optical microscopy (Alpha 300, WiTec) under bright-field illumination. PEI/SLG film thicknesses were determined using profilometry (Dektak 150, Veeco) to measure the height profiles of films transferred from Cu to glass. Height profiles were measured over a scan length of 1500–2000 μm using a 2 μm (diameter) stylus.

### Thermal Annealing of Thin Films

To evaluate the thermal oxidation characteristics of uncoated SLG/Cu, samples without PEI were thermally annealed in a high-temperature vacuum oven (MTI Corp., DZF-6020-HT400P) under ambient air at 250 °C in vacuum for 2 h. To reach 250 °C, the oven was heated at a rate of 10 °C/min to 200 °C, followed by a heating rate of 3 °C/min to 250 °C to avoid significant temperature overshoot. The temperature was then held at 250 °C for 2 h before cooling back to room temperature at a rate of around −10 °C/min.

To evaluate the effect of thermal annealing-induced deformation on the mechanical properties of PEI/SLG, a similar annealing procedure as that of SLG/Cu was conducted. Films were annealed under ambient air in vacuum using the vacuum oven. The temperature of annealing was adjusted from 100 to 200 °C, depending on the desired level of total deformation, and the set temperature was reached using a temperature ramp of 10 °C/min. Once the desired annealing temperature was reached, the temperature was held constant, and the PEI/SLG films were annealed for 2 h. Once annealed, the films were cooled back to room temperature at a rate of 10 °C/min.

### Time–Temperature Analysis of Polymer/Graphene Deformation on Cu

Bright-field optical images were taken of the thermal annealing process in situ using a WiTec Alpha 300 Raman microscope coupled with a Linkam THMS600 thermal stage. Annealing temperatures were chosen between 150 and 250 °C, and PEI/SLG/Cu films were loaded into the thermal stage and heated to the desired annealing temperature at a rate of 10 °C. Imaging was performed at 10× objective magnification in color mode to monitor the deformation of the surface over time, with images taken every 5 min. For annealing temperatures ≥217 °C, additional images were taken at shorter intervals for *t* < 10 min to capture rapid changes in the early stages of film deformation. For each annealing temperature, at least nine distinct spots on the PEI/SLG/Cu surface were imaged to establish statistical significance.

To quantify the extent of deformation in PEI/SLG/Cu with time, images taken of the deformation at each spot on the surface were compiled and processed using ImageJ2 (FIJI) software (available online via the National Institute of Health (NIH)). This image processing and analysis was achieved in several steps, and example images of the processing procedure are provided in Figure S10. First, all images of a given spot were stacked in ascending order with respect to time after reaching the desired annealing temperature (i.e., 0–60 min). The resulting image stack was then aligned to account for thermal expansion-related drift in the position of the film under the objective during annealing. The aligned image stack was cropped to remove the nonoverlapping regions between images. Next, the image at *t* = 0 min was subtracted from all other images in the stack to obtain “baseline subtracted” images depicting the deformed areas in each image. The stack was then converted to 255-grayscale (8-bit), then converted to binary using a threshold of 30. The areal deformation fraction (Δ) was then calculated for each image using
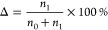
1where *n* is the total number of white (1) or black (0) pixels in the processed image.

### Mechanical Analysis of Poly(ether imide)/Single-Layer Graphene Thin Films

PEI/SLG films were prepared for uniaxial tensile testing using previously reported methods for mm-scale testing of ultrathin polymer/graphene films.^36,41^ Briefly, HDPE frames were created with an internal window of 10 × 15 mm^2^. Thermally annealed PEI/SLG/Cu tensile bars (∼3–6 mm wide, ∼35 mm long) were adhered to the HDPE frame over the internal window using double-sided carbon tape. Once adhered, PEI/SLG was freed from the Cu substrate by chemically etching Cu in ammonium persulfate (APS-100, Transcene Inc.), followed by three DI water rinses for 5 min each. After rinsing, the suspended PEI/SLG specimens were dried in air for several hours before mechanical testing.

Uniaxial tensile tests were performed on thermally annealed PEI/SLG specimens to determine the effect of film deformation on mechanical properties. The tensile tests were conducted using a dynamic mechanical analyzer (Q800 DMA, TA Instruments) in controlled stress–strain mode. The HDPE frame-supported PEI/SLG specimens were loaded into the DMA instrument and clamped along the top and bottom edges of the frame window to secure the films for testing. Once secured, a gap was cut out of the sides of the frame to mechanically decouple the upper and lower halves of the HDPE support. A small preload force (0.001 N) was applied to ensure the films were taut for the measurement. Finally, tensile tests were conducted at 30 °C (isothermal for 2 min) using a force ramp rate of 0.4 N/min and a sampling rate of 2 Hz.

## Results and Discussion

**Figure 1:**
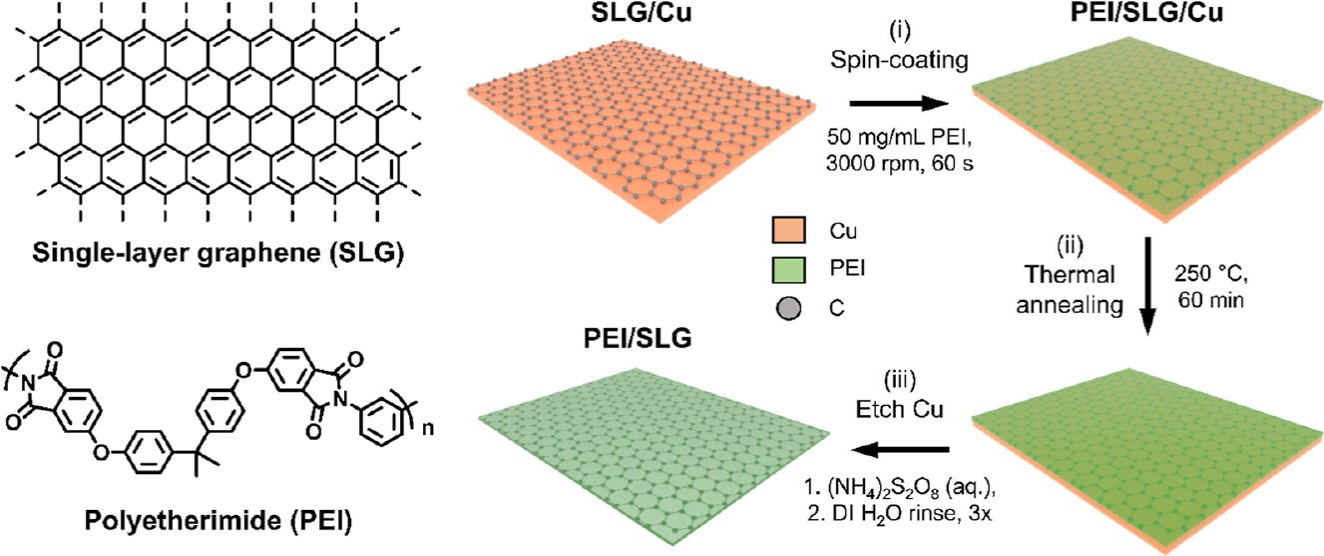
Schematic illustration of the fabrication of PEI/SLG thin films on Cu substrates.

Spin-coating coupled with thermal annealing is a common approach to creating polymer/graphene laminate thin films. While sometimes additional fabrication steps may be needed, these two steps are the basis for most thin-film fabrication processes (e.g., films and membranes).^31−34^ In this study, as a model system to investigate polymer/graphene laminates, we fabricate poly(ether imide)/single-layer graphene (PEI/SLG) thin films on Cu foil (Figure 1). Following our previous reports,^35,36^ a thin layer of PEI was spin-coated over CVD-grown SLG on Cu (SLG/Cu) at submicron thickness (0.63 ± 0.08 μm, Figure S1). Then, PEI/SLG was thermally annealed on Cu for 60 min at 250 °C, which is above the glass transition temperature of PEI (*T*_g_ = 217 °C),^37^ to remove any residual solvent and relieve internal stress from the spin-coating process. Finally, the Cu substrate was removed by wet etching in ammonium persulfate (APS, (NH_4_)_2_S_2_O_8_) to yield free-standing, suspended PEI/SLG films.

**Figure 2:**
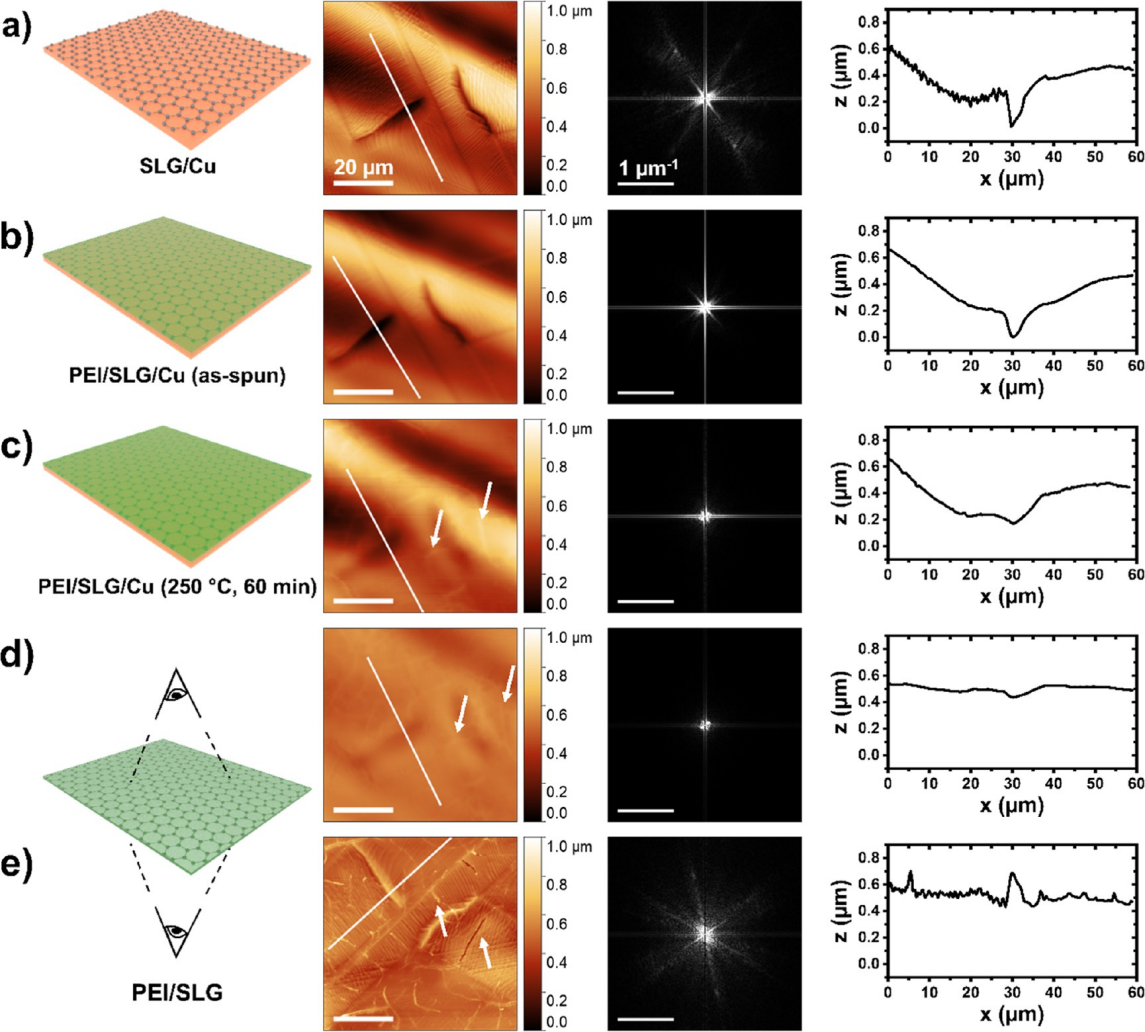
PEI/SLG surface topography at different stages of fabrication. Schematic illustration, AFM height images, and the corresponding 2D FFT and line scans of (a) SLG/Cu (without polymer), (b) as-spun PEI/SLG on Cu (unannealed), (c) PEI/SLG thermally annealed on Cu at 250 °C for 60 min, and (d,e) PEI/SLG after transfer from Cu, as viewed from (d) the PEI and (e) the SLG sides of the film. All images are of the same area at different stages of fabrication. The arrows in (c–e) highlight examples of deformation features that form after thermal annealing and persist through the transfer from Cu. The scale bars correspond to the same type of images.

Typical Cu surfaces are polycrystalline, and the microstructures on the Cu surface are expected to influence the polymer/graphene interface, similar to their influence on SLG during CVD.^38,39^ To reveal the influences of the substrate on polymer thin films, atomic force microscopy (AFM) was employed to monitor the topography of polymer/graphene films throughout the fabrication process. A representative area (Figure 2) showed typical features of the surface at each stage. Additional images are provided in the Supporting Information, showing different areas of the surface at each fabrication stage (Figure S2).

Initially, we determined several key baseline characteristics of the SLG/Cu surface (Figure 2a). First, the Cu surface was polycrystalline and contained a mixture of large and small grains with diverse surface morphological characteristics (Figure S3). Neighboring millimeter-scale grains formed curved grain boundaries (GBs) due to the different orientations of the Cu lattices.^40^ Inside the large grains, smaller, rectangular Cu grains were commonly observed and had lengths and widths of hundreds and tens of microns, respectively. Scanning electron microscopy/electron backscatter diffraction (SEM-EBSD) images showed that the rectangular domains exhibited surface-normal crystallographic orientations of (111) nearly parallel to the free surface (Figure S4). Second, the graphene layer contained sparse multilayer islands (diameter <20 μm), as well as narrow, linear corrugation features (e.g., folds and wrinkles)^9,38^ (width <5 μm, length >20 μm), which occurred randomly over the substrate and were likely introduced during CVD synthesis.^9,38,39^ Finally, the surface topography was largely anisotropic, particularly within twinning boundary domains (Figure S5). The surface exhibited a short-period, terrace-like roughness and a long-period, sinusoidal waviness. In general, the root-mean-squared (rms) surface roughness (*R*_q_) was greatest (≤90 nm) and most highly anisotropic within the rectangular domains, while the arithmetic mean waviness (*W*_a_) was typically ≥100 nm, independent of grain morphology or orientation.

We expected the microstructures of the SLG/Cu surface to translate to the deposited PEI film, given the scale of features on the Cu surface and the submicron thickness of the PEI. AFM height images showed a conformal coating of PEI over SLG/Cu after spin-coating (Figure 2b). Many of the large-scale features of the underlying SLG/Cu were preserved in the PEI layer, especially the largest height variations. However, the fine microstructures of the SLG/Cu substrate were less pronounced, as evidenced by an overall decrease in the rms surface roughness inside the grains. The loss of the fine microstructures is further consolidated by the loss of high-frequency peaks in the 2D fast Fourier transform (FFT) images of PEI/SLG/Cu (Figures 2b and S6).

Thermal annealing of PEI/SLG on Cu caused a dramatic change in the surface morphology. After annealing at 250 °C in air for 2 h, the PEI surface showed unexpected deformations (Figure 2c). All features imprinted on the polymer layer by the underlying SLG/Cu substrate became less pronounced, which was reflected by a decrease in low-frequency peak intensity in the 2D FFT images. To further reveal the changes, the film was adhered to a support ring and transferred from the Cu substrate by etching in APS to yield a free-standing film, then rinsed three times in DI water for 5 min each rinse. Upon transfer, much of the large-scale waviness disappeared in the free-standing film, as shown by the AFM image (Figure 2d). Consequently, the annealing-related deformation became more visible due to the reduction of large-scale waviness.

The SLG side of the film displayed features corresponding to the microstructures of the removed Cu substrate (Figure 2e), as reflected by the return of high-frequency peaks in the 2D FFT image. Additionally, identical deformation features to those found in the polymer layer after annealing were observed on the SLG side of the film. However, the heights of the deformation features at the SLG surface were substantially larger than when viewed on the polymer side of the film, suggesting that the deformation occurred at the SLG/Cu interface.

Our observations indicate that thermal annealing had a pronounced effect on the final microstructures of the polymer/graphene films, which may impact the performance and reliability of the composite. Foremost, the surface features on the Cu substrate (Figure 2) may cause disparities in film thickness up to ∼11% within the roughest areas of Cu due to molding of substrate features into the PEI/SLG interface. The thickness nonuniformity could pose a risk of stress concentration in thinner regions of the polymer film, as well as disrupting the graphene lattice or eventually destroying it. Second, although the upper PEI surface mirrored the substrate microstructures before thermal annealing, the microstructures were partially relaxed during annealing. Conversely, the lower SLG and PEI surface retained the substrate microstructures after annealing and transfer. Finally, the introduction of additional irregular curved line-like deformation features appeared after thermal annealing, which may further impact the mechanical properties of the polymer/graphene heterostructure.

### Deformation-Induced Mechanical Weakening

**Figure 3:**
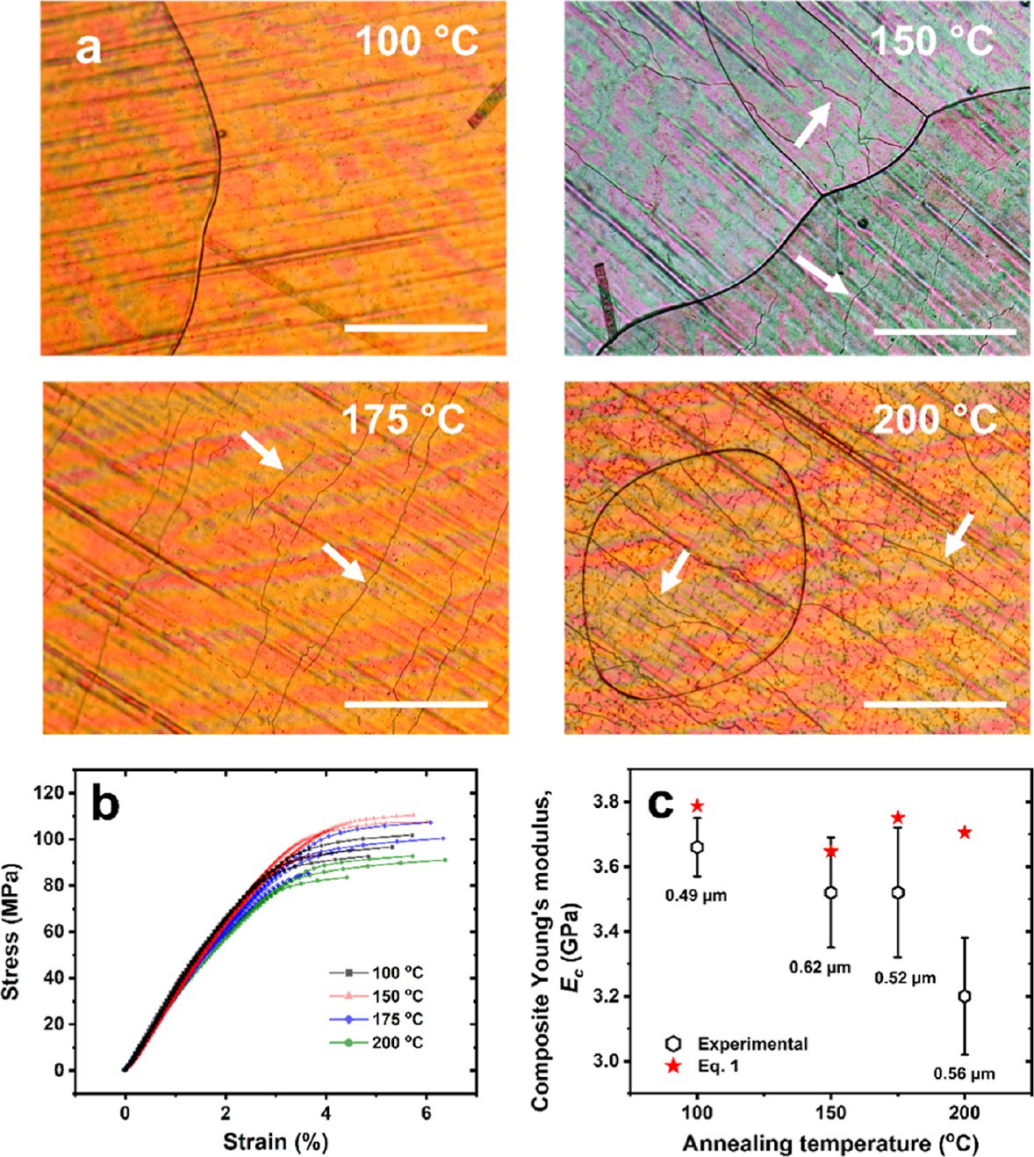
Annealing-induced film deformations and the impact on the effective mechanical properties. (a) Optical images of PEI/SLG/Cu annealed at 100, 150, 175, and 200 °C show a steady increase in the deformations, as visible on the surface and highlighted by arrows. Scale bars, 200 μm. (b) Representative tensile curves of PEI/SLG/Cu annealed at 100, 150, 175, and 200 °C. (c) The average Young’s modulus as a function of the annealing temperature. The corresponding average thickness is provided below each data point, and the theoretical Young’s modulus is calculated using eq 2 based on each thickness and assuming a real SLG and PEI modulus of 1.0 TPa and 3.1 GPa, respectively.^36^

To further investigate the impact of thermal annealing on the mechanical properties of the films, we performed uniaxial tensile tests on PEI/SLG films annealed at temperatures between 100 and 200 °C (Figure 3). This temperature range was chosen because the films had poor survivability when annealed at temperatures above the *T*_g_ of PEI. The tensile specimens were prepared similarly to previous reports.^31,32,35,36,41^ Briefly, precut PEI/SLG/Cu tensile bars were attached to a hollow support frame. Then, the PEI/SLG films were separated from the Cu substrate by etching in APS, followed by rinsing in DI H_2_O. Once dried, the films were tested using a Q800 Dynamic Mechanical Analyzer in a controlled force ramp mode.

As the annealing temperature increased from 100 to 200 °C, the surface density of deformation features increased (Figure 3a). At 100 °C, little to no deformations were observed on the surface. At 150 °C, a small number of deformations became visible, which further increased upon heating to 175 and 200 °C. With an increasing amount of deformations, the tensile properties of PEI/SLG deteriorated, which was marked by a drop in the Young’s modulus from 3.5 ± 0.2 GPa at 150 °C to 3.2 ± 0.2 GPa at 200 °C (Figure 3b,c). The yield at break and the ultimate strength, however, exhibited no clear trend with the annealing temperature.

Typically, the rule of mixtures is used to estimate the effective reinforcement of polymer with graphene.^31,32,42,43^ In this approach, the Voigt upper bound mixing model is used to establish the effective Young’s modulus of graphene (*E*_g_)
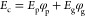
2where *E* and φ are Young’s modulus and volume fraction, and the subscripts c, p, and g denote the composite, polymer, and graphene, respectively. For heterostructures, the volume fraction of individual component, *i*, is given by the ratio of layer thickness to total composite thickness ().^32,36,42^ Thus, *E*_g_ is extracted given a thickness of 0.34 nm for SLG^44^ and knowing the thickness and Young’s modulus of the PEI component. Here we use the average measured thickness for each composite film and a Young’s modulus of 3.1 GPa for PEI, which we measured previously.^36^ Importantly, this approach allowed us to account for the effect of variable thickness on the overall reinforcement.^36,42^

We found that *E*_g_ steadily decreased as the annealing temperature increased. However, these *E*_g_ values most likely do not reflect the true modulus of SLG. According to the shear-lag theory, *E*_g_ depends on the aspect ratio of SLG, its orientation with respect to the polymer matrix, and the strength of the polymer–graphene interface.^43^ Considering the high aspect ratio and ordered geometry of our films, the change in *E*_g_ for PEI/SLG annealed at temperatures of 100–200 °C should be closely related to the strength of the polymer/graphene interface. Thus, the rapid drop-off of *E*_g_ as the annealing temperature approaches 200 °C suggests that high-temperature annealing has a detrimental effect on the interfacial strength. This phenomenon may be rationalized by the increased deformation observed with the increasing annealing temperature. Thus, the annealing process appears to have a negative impact on the effective reinforcement between polymer and SLG by introducing undesirable deformation features.

The degraded mechanical strength upon thermal annealing may provide an explanation for discrepancies between the effective reinforcement of polymer and graphene. Even though graphene is known to have a high Young’s modulus of ∼1.0 TPa, the effective graphene modulus found for polymer/graphene heterostructures in many studies is much lower than the expected values based on the rule of mixtures.^31,32^ For example, Liu et al. found an effective *E*_g_ of 360 GPa using the rule of mixtures for polycarbonate (PC)/SLG laminates, which were prepared by stacking multiple layers of PC/SLG and then hot-pressing at 150 °C.^31^ Wang et al. found an effective *E*_g_ of 810 GPa for similarly stacked PC/SLG heterostructures.^32^ PC typically has a *T*_g_ of ∼150 °C. Considering the discrepancy between *E*_g_ values in these two similar studies, we suspect that thermal processing near the *T*_g_ of PC may have impacted the properties of the composites, similar to this work. Thus, an in-depth understanding of the deformation mechanism is called for and may help guide future protocols for thin-film composite processing.

### Mechanism of Thermally Induced Deformation

We hypothesized that the cause of the deformation in PEI/SLG during annealing was likely related to substrate oxidation, given the known susceptibility of graphene-coated Cu to interfacial oxidation, even at ambient temperatures.^45−51^ It is reported that defective sites in graphene provide vulnerable points for the generation of oxygen radicals from water decomposition that penetrate the film at grain boundaries and oxidize the Cu substrate.^45,47^ The susceptibility of SLG/Cu used in this study to surface oxidation was verified using AFM and SEM–EDS (Figure S7). When annealed at 250 °C for 2 h, SLG/Cu without PEI showed enhanced roughness across the entire surface. Additionally, SEM-EDS maps showed an increased density of O atoms and a decreased density of Cu atoms in deformed areas after annealing, confirming the presence of Cu_2_O/CuO.

**Figure 4:**
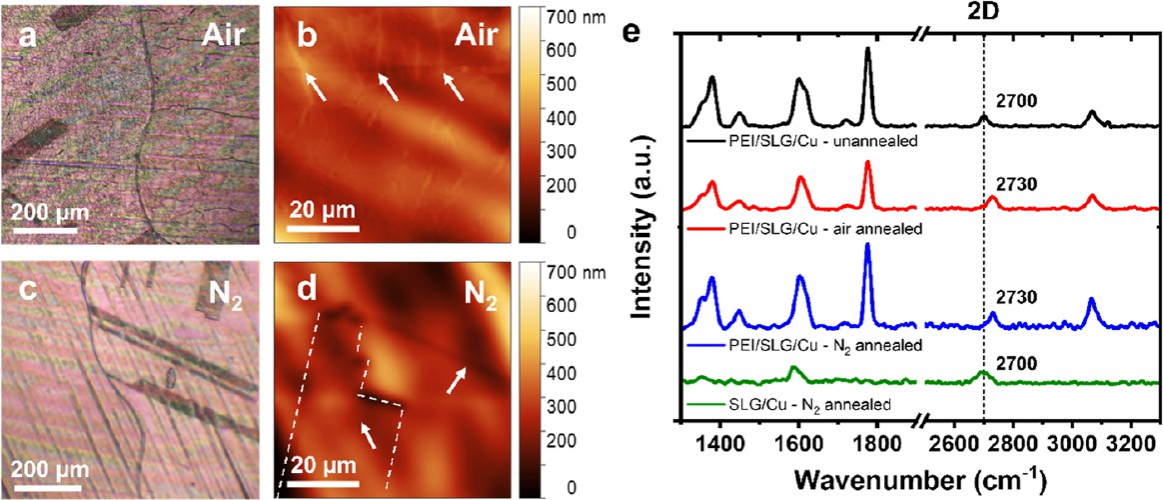
Thermal annealing-induced deformation of PEI/SLG on Cu under a controlled atmosphere. Representative (a,c) optical and (b,d) AFM micrographs of PEI/SLG/Cu after annealing at 250 °C for 60 min (a,b) in air and (c,d) under N_2_. (e) Raman spectra of the surface before and after thermal annealing in N_2_ and air. Before annealing, the 2D peak position of SLG is ∼2700 cm^–1^ (dashed line). After annealing in either N_2_ or air, the 2D peak position blue-shifts to ∼2730 cm^–1^, indicating compressive stress. Without polymer, the 2D peak position does not shift and remains at 2700 cm^–1^.

To probe the role of O atoms in the deformation mechanism for PEI/SLG/Cu, additional experiments were conducted under controlled atmospheric conditions. Optical micrographs showed a large degree of deformation in the films annealed in moist ambient air (relative humidity (RH) ≈ 40–60%) (Figure 4a). In contrast, no deformation was observed on the surface of PEI/SLG/Cu annealed at the same temperature for the same time duration in an oxygen- and water-free atmosphere (≥99.999% N_2_) (Figure 4c). This control experiment suggests that oxygenated gases likely play a role in the deformation of PEI/SLG on Cu, as in similar cases with uncoated SLG/Cu.^45,47,51^ However, our ability to observe the deformation by optical microscopy hinged upon the optical contrast between CuO and Cu.^45,47,50,51^ Therefore, further AFM imaging was performed to confirm the result.

AFM images of PEI films annealed in air contained out-of-plane ridgelines, which corresponded to the deformation observed in optical micrographs (Figure 4a,b, arrows). Conversely, for films annealed under high-purity N_2_ (≥99.999%), no deformations were observed using AFM. In fact, many large-scale features of SLG/Cu (e.g., GBs, twinning domains) remained visible on the polymer surface when annealed under N_2_ (Figure 4c,d, arrows). The comparison between films annealed in air and N_2_ indicates that oxygenated gases are required for PEI/SLG on Cu to permanently deform upon thermal annealing.

The size and shape of the oxidation features on the surface of SLG/Cu were distinctly different from those on PEI/SLG/Cu (Figure S7). The difference suggests that even though substrate oxidation is a key component of the deformation mechanism, the process is also strongly influenced by the polymer layer. Furthermore, for PEI-coated SLG/Cu, the observed deformation patterns formed ring-like networks across the surface of the film/substrate, which persisted throughout annealing. This deformation pattern contrasts with a previous report,^45^ which showed the delineation of oxidative deformations on SLG/Cu at temperatures and oxidation times similar to those used herein. Instead, the deformation patterns observed for PEI/SLG/Cu were more reminiscent of buckling delamination patterns typical of the adhesive failure of compressively stressed laminate materials.^48,52^

For our material, changes in the strain state of the SLG graphitic structure were used to elucidate more about the deformation mechanism. Raman spectroscopy confirmed a compressive strain in SLG after thermal annealing, showing a blue shift of the 2D peak from ∼2700 to ∼2730 cm^–1^ (Figure 4e). The corresponding strain state of SLG after annealing was determined by^53−56^
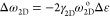
3where Δω_2D_ is the change in 2D peak position, ω_2D_^o^ is the initial 2D peak position before annealing, γ_2D_ is the biaxial Grüneisen parameter, and Δε is the change in biaxial strain of SLG. Given a Grüneisen parameter of 2.7 for biaxially compressed SLG,^55^ the observed blue shift after annealing corresponded to an induced compressive strain of ∼0.21%. Importantly, an identical change in compressive strain was observed in SLG for films annealed under both air and N_2_, indicating that the SLG undergoes compression regardless of Cu oxidation. Furthermore, the Raman spectrum of SLG/Cu without PEI showed no blue shift of the 2D peak after annealing under N_2_, suggesting that PEI is the source of the induced compression in SLG during annealing for PEI/SLG/Cu. It is worth mentioning that the blue shifting of the 2D peak is also an indicator of hole doping and is commonly observed as a response of graphene to chemical interactions with its environment, including adsorption of H_2_O/O_2_ and exposure to PMMA chains.^13,19,20^ However, the blue shift of 30 cm^–1^ here is much larger than typically seen during environmental doping events. Thus, the mechanical straining of SLG is more likely to be the driving force behind the blue-shift in PEI/SLG during thermal annealing.

We suspected that the annealing-induced strain in SLG was linked to the release of internal stress from PEI during annealing. For spin-coated polymer films, a time-dependent internal stress develops due to constrained shrinkage of the polymer on the substrate during spin-coating.^25^ This internal stress is related to the evolving mechanical properties of the film via Maxwell’s model^25^
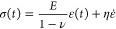
4where *E* is Young’s modulus, ν is Poisson’s ratio, ε(*t*) is the time-dependent strain (due to shrinkage), η is the film viscosity, and  is the strain rate. Depending on spin-coating speed and solvent evaporation rate, polymer chains may adopt nonequilibrium conformations and orient in preferential directions.^57,58^ These nonequilibrium orientations create internal stress in the polymer, which becomes trapped due to the quenching of chains under rapid solvent evaporation.^26^

Under the Maxwellian model, the time scale for internal stress relaxation is proportional to the ratio of viscosity to shear modulus.^25^ Given that spin-coating is employed for the deposition of PEI on SLG/Cu, we anticipated that the PEI layer of our films would exhibit a certain degree of internal stress. Dynamic mechanical thermal analysis (DMTA) showed that the PEI/SLG films had a *T*_g_ of 218–221 °C (Figure S8). While in the glassy state, the relaxation modulus of PEI/SLG remains high, and thus the time scale for relaxing internal stress will be much larger than the time scale of thermal annealing. However, upon glass-to-rubber transition, the relaxation modulus of PEI rapidly decreases. As a result, the time scale for stress relaxation is dramatically reduced, and the loss of internal stress by the conformational rearrangement of polymer chains occurs rapidly. The loss of this internal stress during thermal annealing on Cu is accompanied by a reduction in tensile strain (eq 4). Consequently, the PEI film is expected to undergo in-plane shrinkage within the time-scale of the annealing (i.e., ∼1–2 h) when annealed above *T*_g_.

To test our hypothesis, an analogous experiment was conducted using PEI thin films spin-coated and thermally annealed at 250 °C for 2 h on glass. AFM images before and after annealing showed that the PEI surface roughness (*R*_tm_) increased significantly from 5 ± 2 to 29 ± 9 nm following thermal annealing (Figure S9). The substantial roughening of the PEI surface is likely a consequence of changes in the microstructure of PEI induced by thermal treatment, such as by the rearrangement of polymer chains and shrinkage of the matrix. Relating this to the thermal annealing of PEI/SLG, the compressive strain in SLG found by Raman spectroscopy after annealing might be explained by the transfer of stress from PEI to SLG upon thermal relaxation of the polymer. However, further measurements probing the stress state of spin-coated polymer layers, like PEI, will be necessary to confirm this hypothesis.

**Figure 5:**
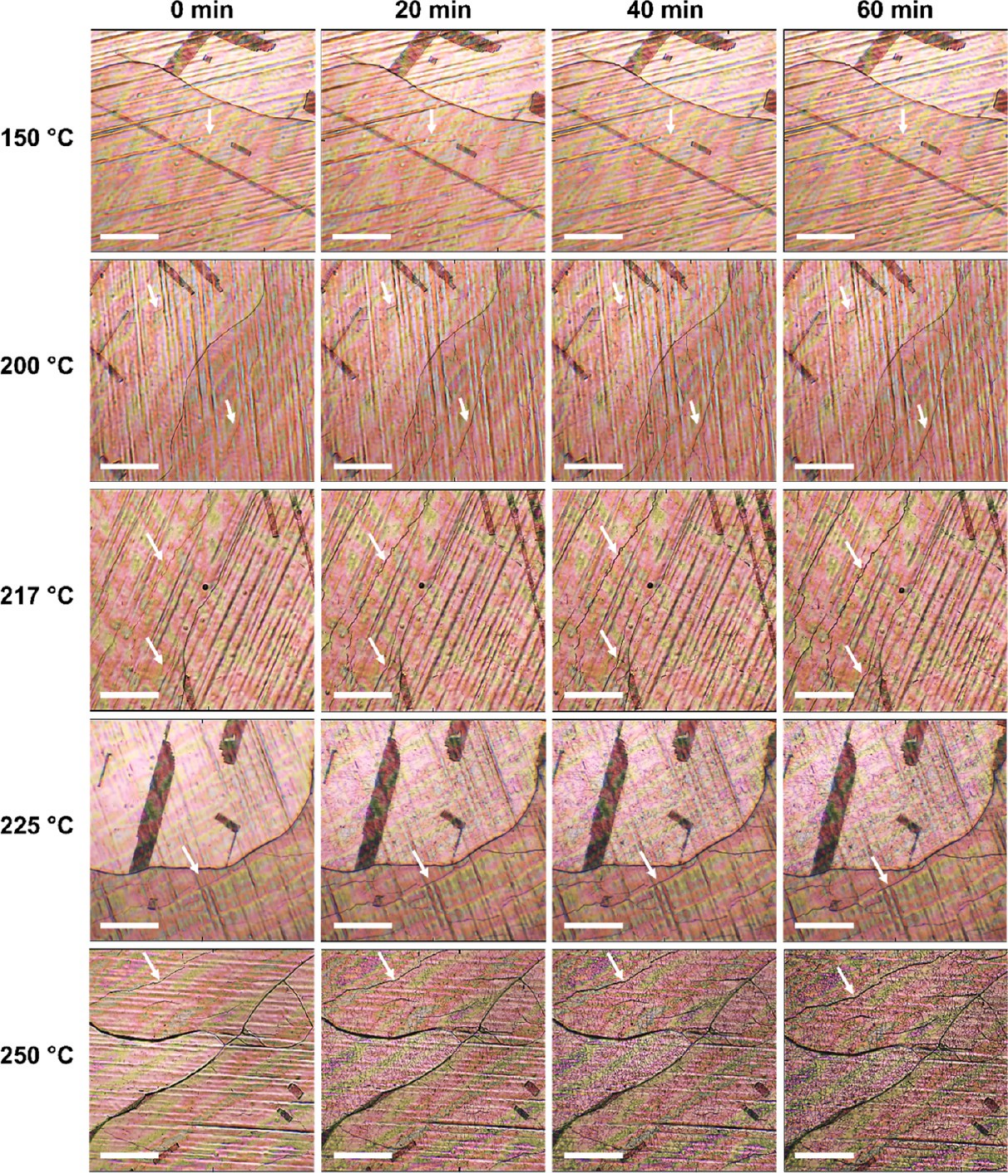
Optical micrographs show the time–temperature dependence of Cu oxidation beneath PEI/SLG thin films. Below ∼217 °C (the *T*_g_ of PEI),^37^ little deformation is observed. Above 217 °C, widespread deformation is observed within 60 min of annealing. White arrows highlight the evolution of select deformation features. Scale bars, 200 μm.

To gain further insight regarding the role of PEI in the deformation mechanism, kinetic studies were performed using in situ optical microscopy to analyze the time evolution of the deformation. Time-lapse imaging of the thermal annealing process confirmed that the deformation of PEI/SLG/Cu is highly temperature-dependent (Figure 5). Moreover, widespread deformation of the film was not observed until the annealing temperature exceeded the *T*_g_ of PEI (218–221 °C, Figure S8). By contrast, earlier reports have shown the onset of oxidation for uncoated SLG/Cu at much lower temperatures (∼180 °C).^45,50^ Thus, PEI appears to inhibit oxidative deformation of SLG/Cu while in the glassy state. Instead, the onset of deformation for PEI/SLG/Cu is more closely linked to the glass-to-rubber transition of the polymer during thermal annealing.

To verify our finding, we quantified the time evolution of the film/substrate deformation from the in situ optical micrographs. To be thorough, images were taken of at least nine distinct spots on the PEI/SLG/Cu surface at regular intervals during annealing, and a total of seven different annealing temperatures from 150 to 250 °C were investigated. The extent of deformation in each image was determined by processing the images using ImageJ to find the relative change of each surface with annealing time. By subtracting the initial image from the images at each interval, the areal deformation fraction (Δ), defined as the ratio of deformed surface to total surface in the image, was determined (see eq 1 in the Experimental Section and Figure S10 for details). For reference, fully processed versions of the images in Figure 5, which highlight the deformations in each image, are provided in the Supporting Information (Figure S11).

**Figure 6:**
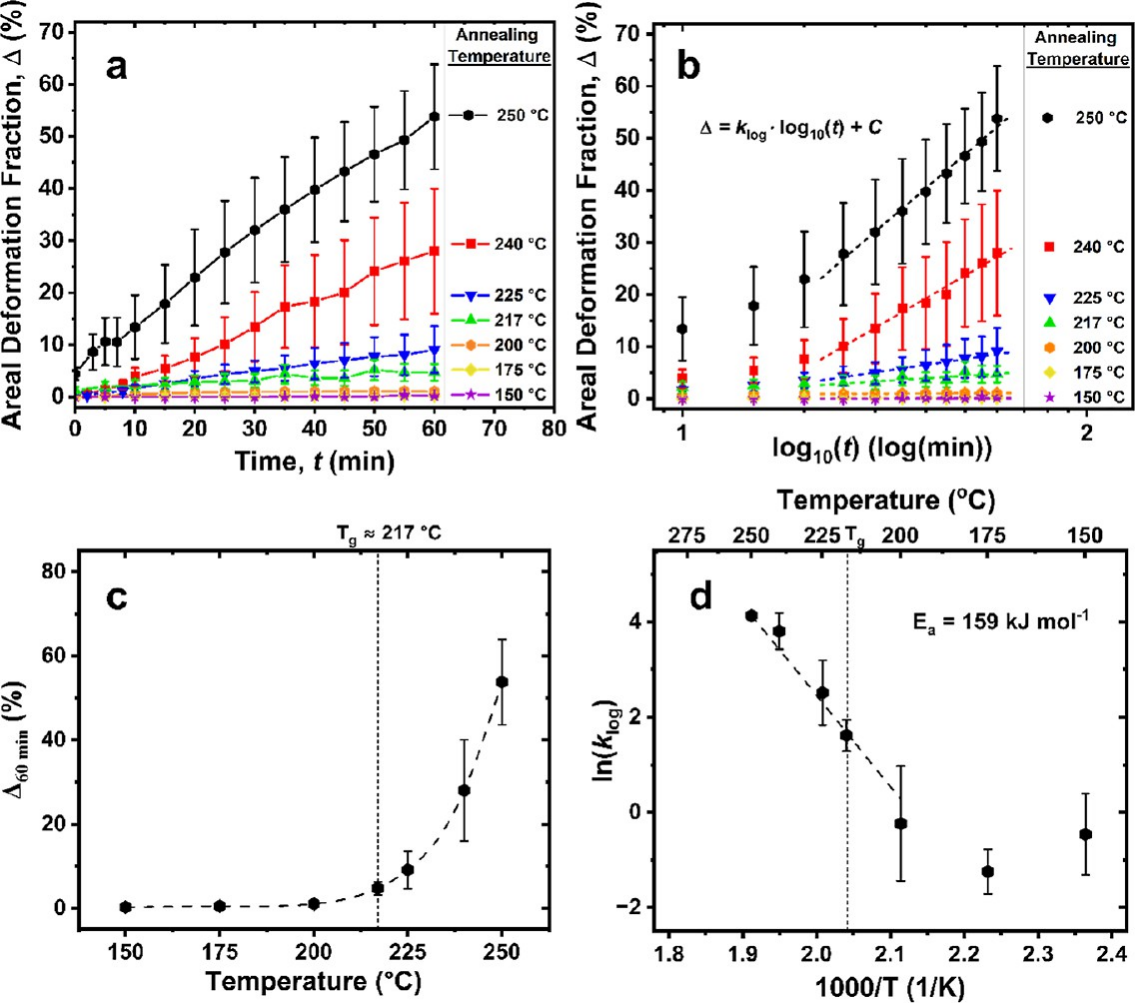
Kinetics of film deformation. (a) Fraction of deformed areas as a function of time and temperatures over different Cu crystal facets. (b) Data from panel (a) represented in log scale of time for kinetic fitting because of the logarithmic rate law of thin oxide film formation.^59^ (c) The average areal deformation fraction of PEI/SLG surfaces after 60 min of annealing as a function of temperature. (d) Arrhenius plot of the rate constant as a function of the inverse annealing temperature, yielding activation energy (*E*_a_) for the deformation process above *T*_g_ of PEI (dashed line region).

Overall, our quantitative analysis corroborated our qualitative observations. The average Δ after 60 min (Δ_60min_) remained <5% of the sample surface on average at annealing temperatures up to 217 °C (Figure 6a,c). Beyond 217 °C, Δ_60min_ began to increase dramatically from 9 ± 4% at 225 °C to 54 ± 10% at 250 °C. However, the total extent of oxidative deformation was notably lower than that reported previously for SLG/Cu without a polymer coating. For example, Kim et al.^45^ found >80% surface oxidation for SLG/Cu annealed at 240 °C in a similar atmosphere. Additionally, the shape of SLG/Cu oxidation features in Kim’s report is markedly different than those on PEI/SLG/Cu herein. Thus, PEI appears to mediate the oxidation of SLG/Cu to varying degrees below and above the *T*_g_ of PEI, which supports our hypothesis that widespread deformation is activated by the glass-to-rubber transition of PEI.

From the kinetics data, we performed an Arrhenius analysis of the deformation process to obtain greater insight into the deformation mechanism. To obtain the kinetic constant (*k*) at each annealing temperature, the kinetics data in Figure 6a was converted to a logarithmic scale in the time domain due to the expected logarithmic rate law for the formation of thin oxide films (Figure 6b).^45,59^ Here, the kinetics data was similarly fit using linear regression in a logarithmic time scale. From the logarithmic kinetics, we obtained an activation energy (*E*_a_) of ∼159 kJ/mol (Figure 6d) following the Arrhenius relationship, which is approximately double that typically reported for Cu oxidation at temperatures of 150–250 °C (∼75–85 kJ/mol).^60,61^ It is worth noting that there appeared to be a difference in the deformation rates of the films over different areas of the substrate, possibly related to the film/substrate interfacial interaction. The oxidized Cu regions induced defects and damages to graphene, due to volume expansion mismatches between graphene and Cu, eventually degrading and deteriorating the nanostructures (Figure S7). However, we did not have enough EBSD data to fully correlate different crystallographies to the observed deformation rates.

## Conclusions

We have found that thermal annealing in air or vacuum degraded the mechanical properties, including tensile strength and Young’s modulus, of polymer/graphene laminate films. The weakened mechanical properties were associated with changes in the micro- and nanostructures in the thin films. The structural deformation was tracked using microscopy, and the deformation was linked to the polymer glass transition. Above the glass transition temperature (*T*_g_) of the polymer, the deformation rate was significantly higher. Raman spectroscopy revealed that after annealing above the *T*_g_, the 2D peak of SLG blue-shifted ∼30 cm^–1^, indicating a compressive strain in SLG regardless of the atmospheric composition. This compressive strain appeared only when a polymer layer was present, which suggested that the deformation likely resulted from the release of internal stress in the polymer matrix during thermal annealing. The Cu substrate also played an important role in film deformation, where the presence of oxygenated species resulted in Cu oxidation, and therefore, film deformation. Conversely, without oxygenated species, no deformations were observed, indicating that Cu oxidation was responsible for the permanent deformation of PEI/SLG films. The oxidized Cu destroyed the graphene structure, weakening the graphene nano-structure and the entire bilayer structure. The results herein can be extended to other laminate polymer/graphene thin films and may guide the improvement of film fabrication processes. A controlled, inert atmosphere during thermal annealing can minimize unnecessary substrate oxidation to reduce fabrication-related defects in polymer/graphene films. By purging the environment with an inert gas such as N_2_, or removing oxygenated gases, the resulting oxidation can be avoided. We expect further improvement of the fabrication processes for polymer/graphene heterostructures, which will enable more robust and reliable structures with predictable performances and facilitate the commercialization of these materials.

## Supporting Information Available

PEI/SLG thickness profile, AFM images of PEI/SLG at different stages of fabrication, SEM and SEM-EBSD micrographs of SLG/Cu, AFM analysis of the SLG/Cu surface, AFM amplitude images and 2D FFT analysis of PEI/SLG at different stages, oxidation characteristics of SLG/Cu, DMTA measurement of PEI/SLG, AFM surfaces of PEI-on-glass, optical image processing procedure for kinetic analysis, example binary images produced by image processing (PDF)
