# Supplementary material for: Copper Oxidation-Induced Nanoscale Deformation of Electromechanical, Laminate Polymer/Graphene Thin Films during Thermal Annealing: Implications for Flexible, Transparent, and Conductive Electrodes
Source: ACS Appl Nano Mater. 2024 Dec 12;7(24):28829–40. doi: 10.1021/acsanm.4c06372 (PMC11686465; doi:10.1021/acsanm.4c06372)
Supplement: Supplementary file 1 — an4c06372_si_001.pdf [file an4c06372_si_001.pdf]

SUPPORTING INFORMATION

Copper Oxidation-Induced Nanoscale Deformation of Electromechanical, Laminate Polymer/Graphene Thin Films during Thermal Annealing: Implications for Flexible, Transparent, and Conductive Electrodes

Zacary L. Croft,[1] Oscar Valenzuela,[1] Connor Thompson,[1] Brendan Whitfield,[1] Garrett Betzko,[2] and Guoliang Liu[1,2,3,4*]

[1]*Department of Chemistry, Virginia Tech, Blacksburg, VA 24061, USA*

[2]*Division of Nanoscience, Academy of Integrated Science, Virginia Tech, Blacksburg, VA 24061, USA*

[3]*Macromolecules Innovation Institute, Virginia Tech, Blacksburg, VA 24061, USA*

[4]*Dept. of Chemical Engineering, Department of Materials Science and Engineering, Virginia Tech, Blacksburg, VA 24061, USA*

*Corresponding authors: gliu1@vt.edu

Supporting Experimental Information

Figures S1 provides thickness profilometry measurements of polyetherimide/single-layer graphene (PEI/SLG) on glass. Figure S2–S7 and S9–S11 provide additional AFM, SEM, and optical micrograph data to support surface changes observed due to thermal annealing. Figure S8 provides dynamic thermal-mechanical analysis of PEI/SLG demonstrating key thermal transitions.

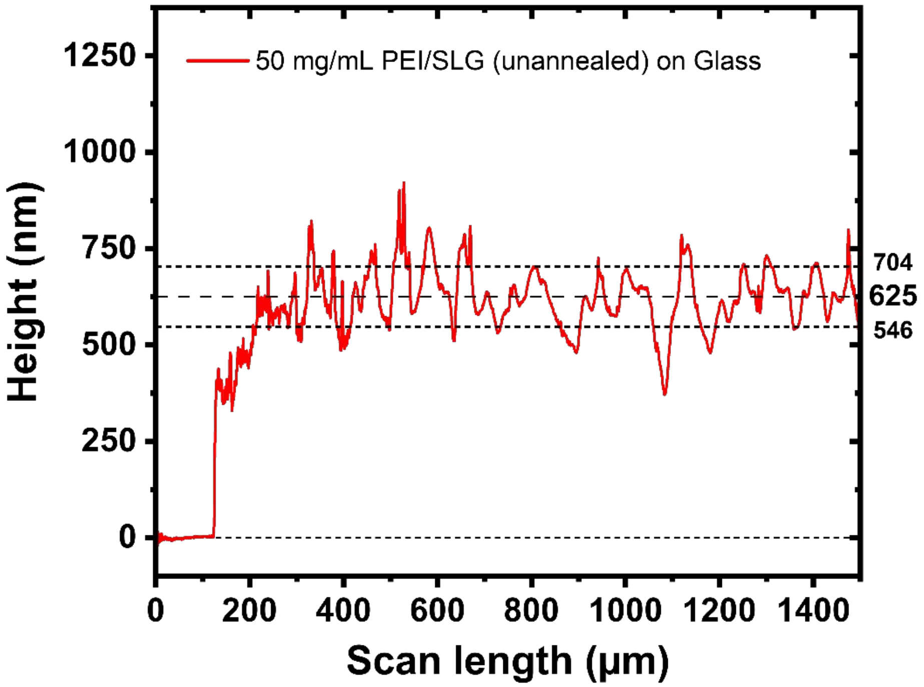

Figure S1. Example height profile of a PEI/SLG film on glass for thickness determination. A 50 mg/mL PEI/$CHCl_3$ solution was spin-coated on SLG/Cu at 3000 rpm for 60 s under N_2. Following the transfer of PEI/SLG from Cu to a clean glass slide *via* wet-chemical etching, the film thickness was determined from, at minimum, five height profiles using the smooth glass surface (RMS surface roughness, R_q < 5 nm) as a zero-point reference. The mean thickness was found to 0.63 ± 0.08 μm, where the uncertainty in the mean was taken as R_q of the film within the measured area.

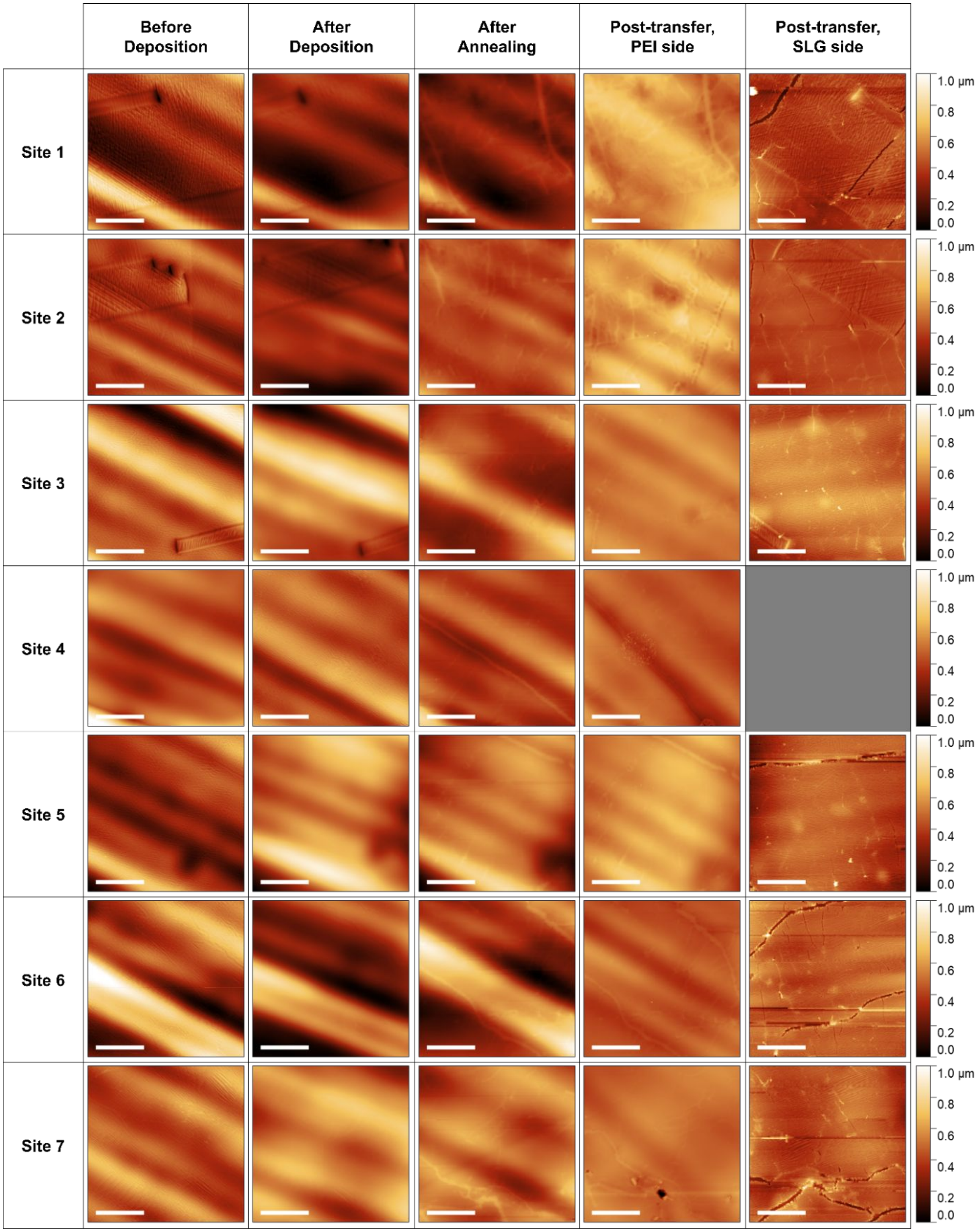

Figure S2. AFM images of PEI/SLG on and off polycrystalline Cu at different stages of fabrication (e.g., before/after polymer deposition, thermal annealing, and transfer).

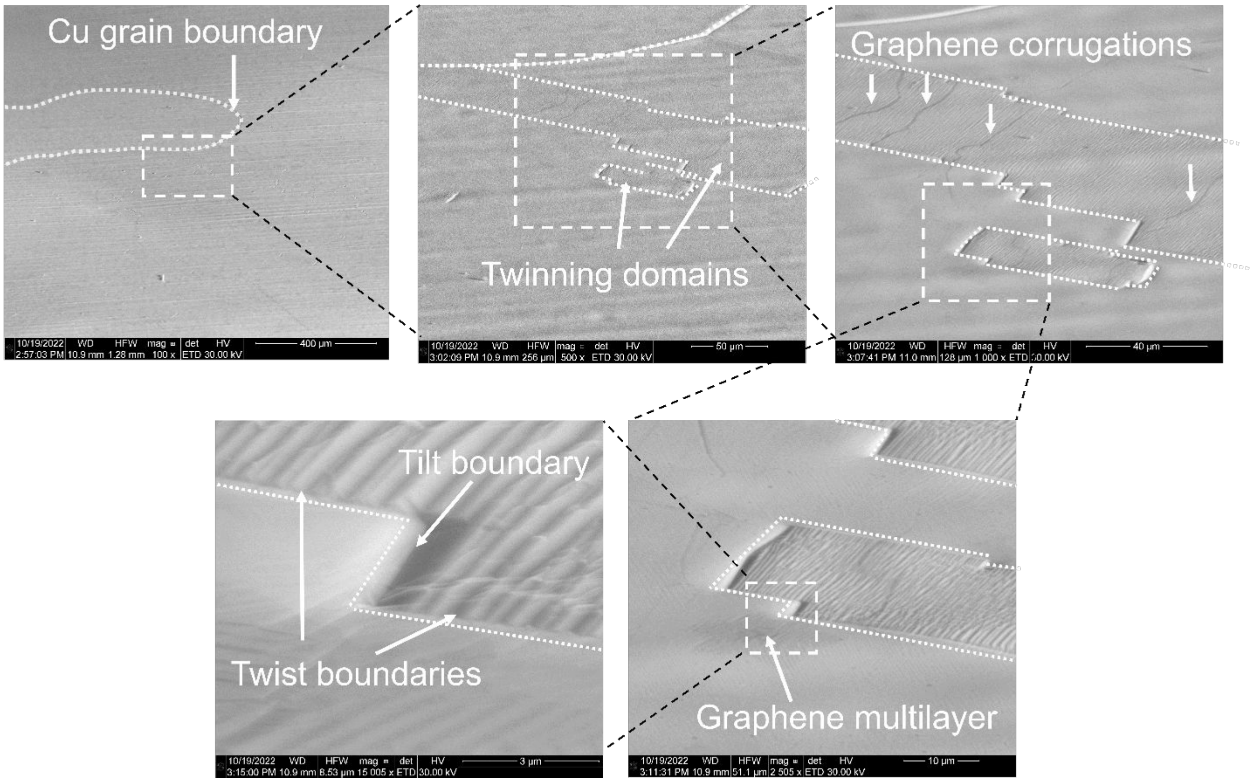

Figure S3. SEM micrographs of SLG on small-grain, polycrystalline Cu show a heterogeneous surface containing several notable Cu features, including a curved grain boundary, several twinning boundary domains ("Strips"), and graphene folds (mainly over strips).

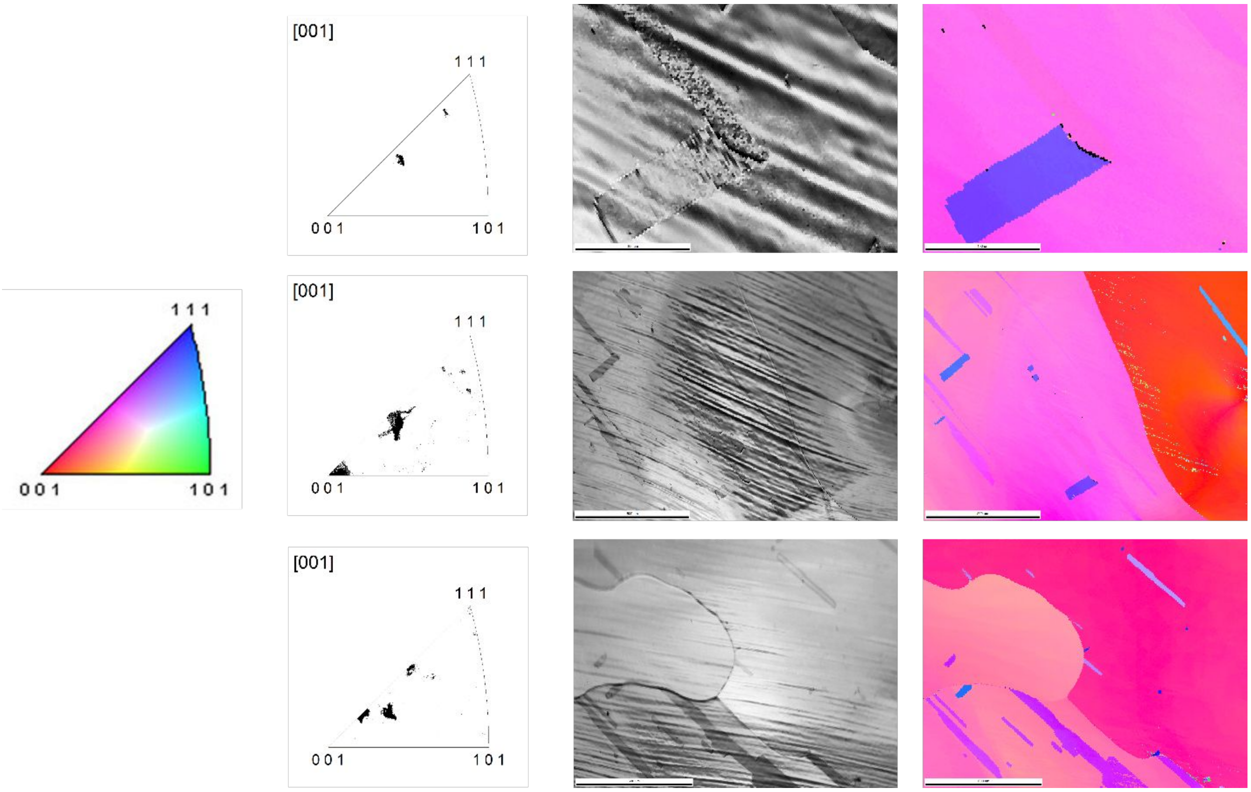

Figure S4. SEM-EBSD micrographs of small-grain, polycrystalline Cu/SLG for the determination of Cu crystal facets. Rectangular strip domains are mainly Cu(111) as seen in the color map, with the larger, curved grains being comprised of Cu(001) and a mixture of Cu(001) and Cu(111) crystallographic orientations.

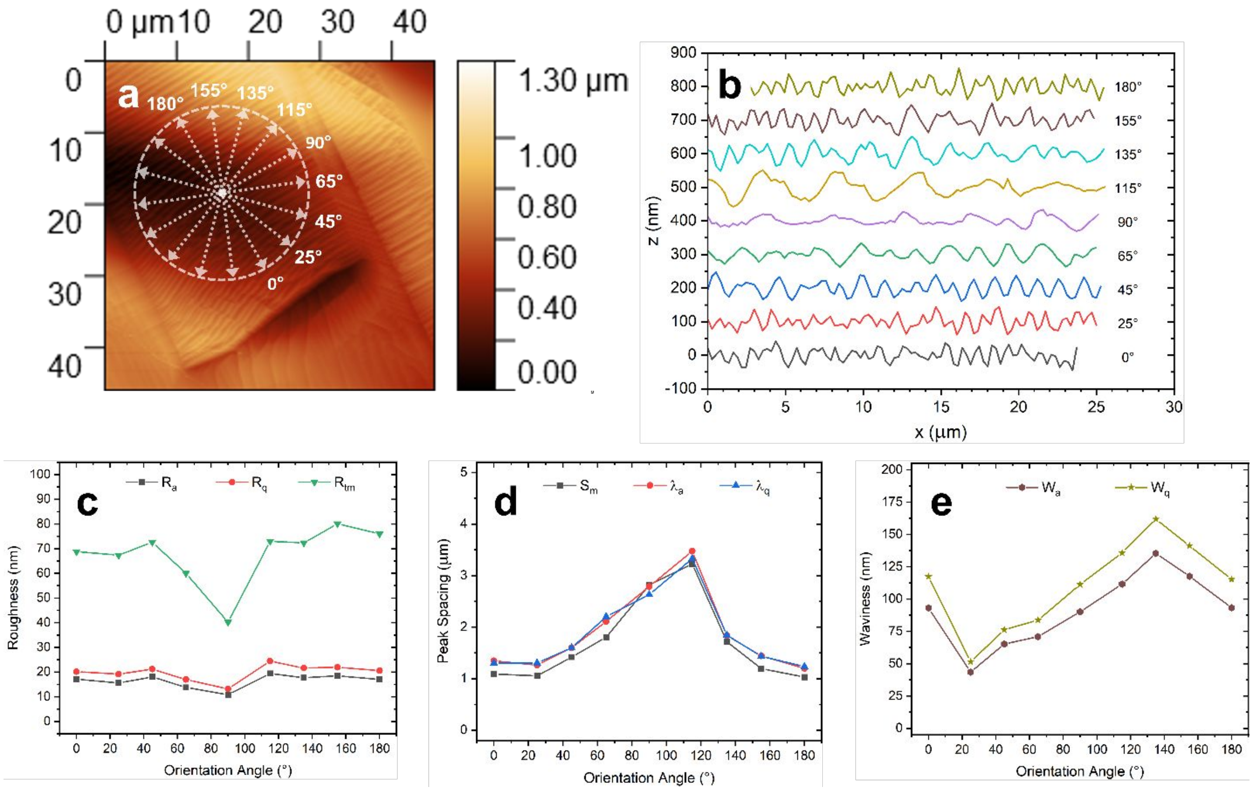

Figure S5. (**a**) AFM image of a representative strip domain of copper substrate. Plot of copper surface roughness along different radial angles. Plots of roughness, peak spacing, and waviness set against orientation angle. AFM analysis of one SLG/Cu(111) twinning boundary domain ("Strip domain") surface from Figure 2 of the main text. (**b**) Height profiles of the strip domain at different orientation angles from 0 to 180° demonstrate that the surface topography is anisotropic. Arithmetic, RMS, and (except for waviness) mean values for (**c**) roughness (R_a, R_q, and R_m), (**d**) peak spacing (λ_a, λ_q, and S_m), and (**e**) waviness (W_a and W_q) vary with orientation angle, reflecting the anisotropy of the Cu strip domain.

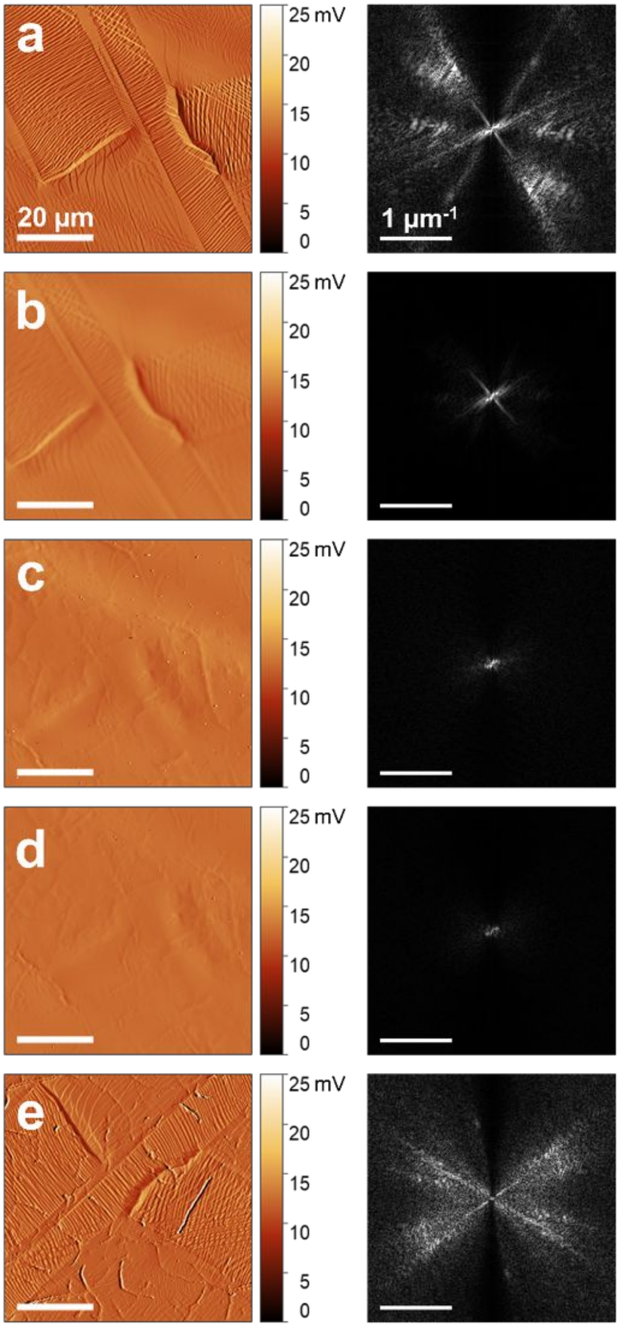

Figure S6. Corresponding real and inverse space amplitude images of the same spot in Figure 2 (main text) at different stages of fabrication. The use of scan amplitude for constructing the images allows for better visualization of the microstructural changes in PEI/SLG caused by processing. (**a**) SLG/Cu before deposition shows high and low-frequency peaks in the 2D FFT images, corresponding to step-terraces and GBs/corrugation, respectively. (**b**) PEI/SLG/Cu shows a loss

of high-frequency surface texture (step-terraces) and retention of low-frequency textures (GBs/corrugation). (**c**) After annealing, the low-frequency intensity in the 2D FFT is diminished, indicating the loss of GB texture. (**d**) After transfer, further low-frequency intensity is diminished due to the decrease of surface corrugations. (**e**) The opposite SLG surface shows the retention of high and low-frequency microstructures from Cu observed before PEI deposition.

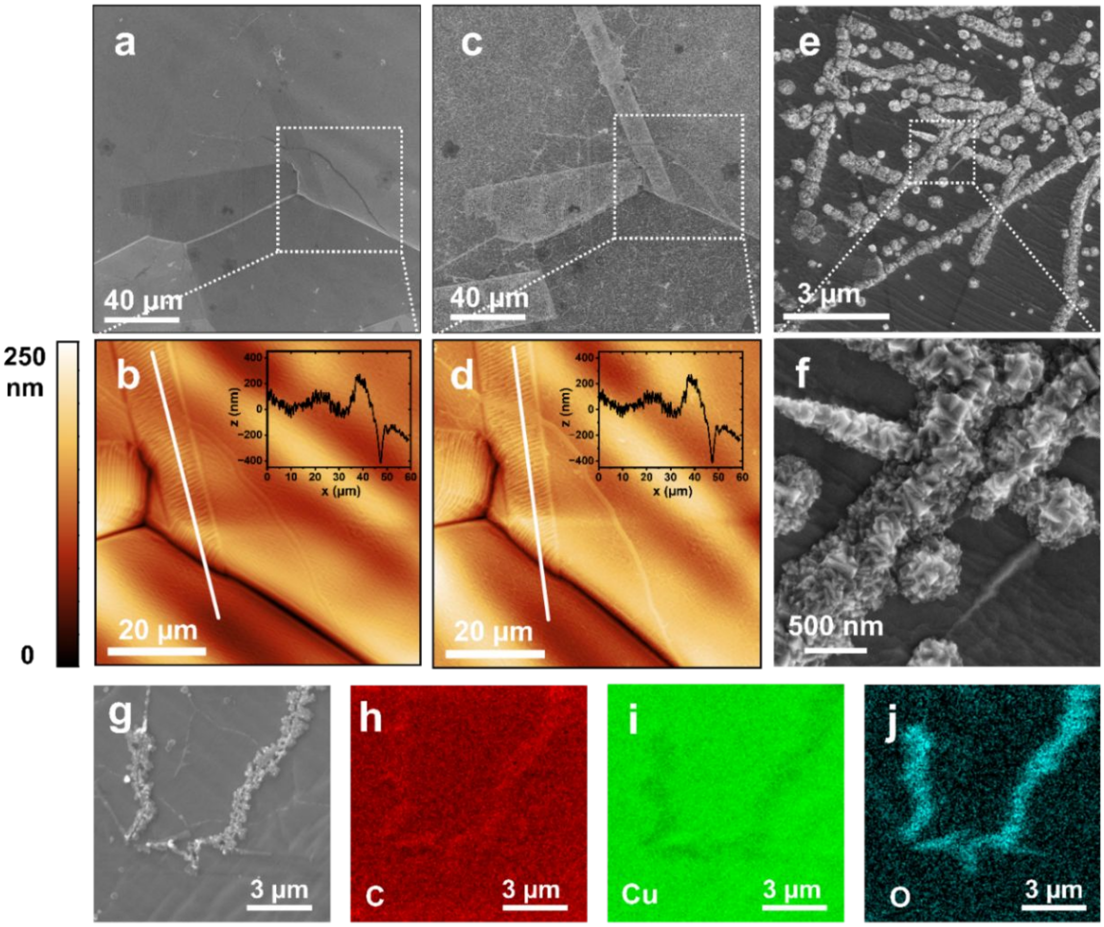

Figure S7. SEM and AFM images of thermally treated copper, including copper oxide structures. EDS maps of different elements on the surface of thermally treated copper. Oxidation characteristics of uncoated SLG/Cu thermally treated at 250 °C for 2 h. (**a**,**b**) SEM and AFM micrographs of the SLG/Cu surface before thermal treatment show typical surface features, including twinning boundary domains, Cu grain boundaries, and large Cu grains. (**c**,**d**) The same SLG/Cu surface after annealing exhibits enhanced charge contrast between Cu grains and increased surface roughness, indicating isotropic oxidation of the surface due to thermal treatment. (**e**,**f**) High-resolution SEM micrographs of the partial surface oxidation of SLG/Cu show 1D and 0D features comprised of "flower-like balls",[1] a morphology indicative of CuO particles formed following the penetration of atomic oxygen at SLG defect sites. (**g**-**j**) SEM-EDS micrographs show

areas of surface deformation on SLG/Cu that display increased O atom density and decreased Cu atom density, consistent with the formation of copper oxides.

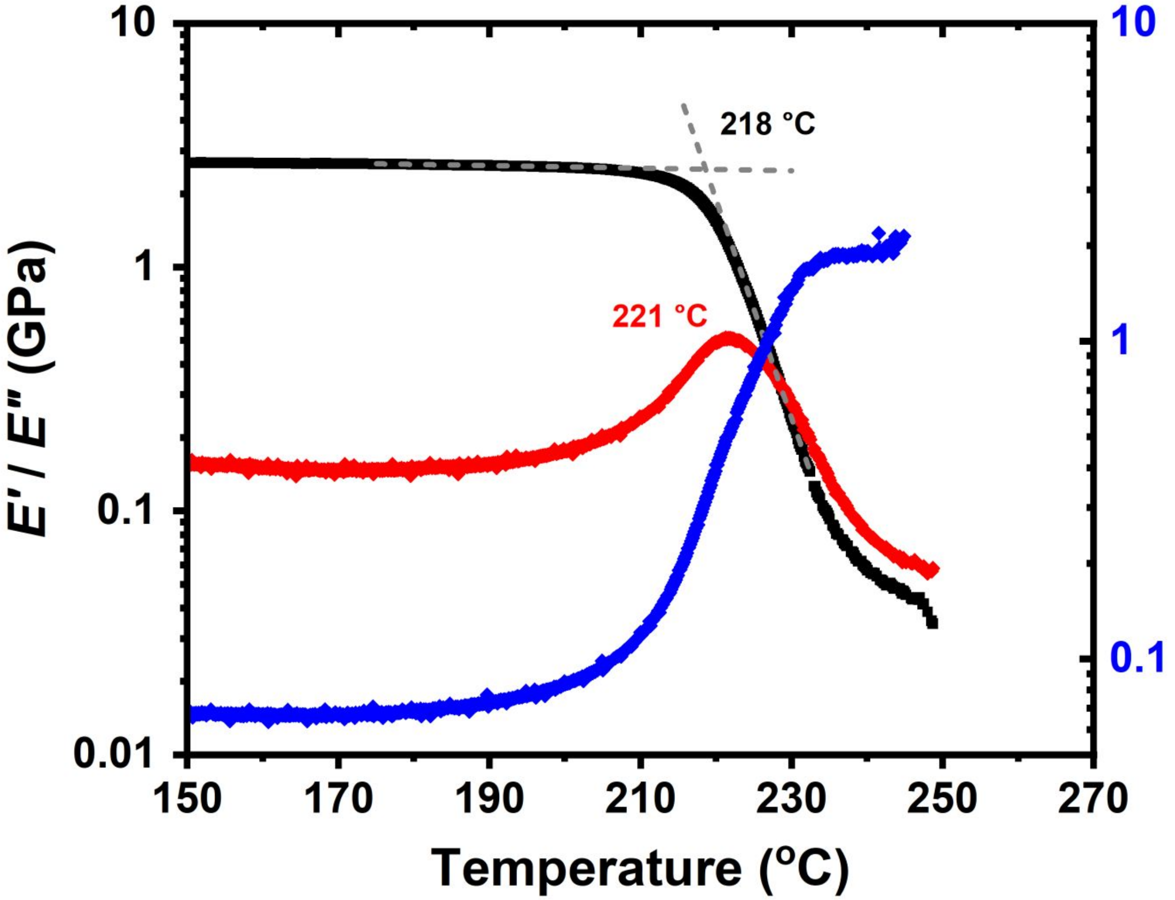

Figure S8. Dynamic mechanical thermal analysis (DMTA) traces show the storage modulus (E', black), loss modulus (E'', red), and $\tan\delta$ (E'/E'', blue) of PEI/SLG as a function of temperature within the range of annealing temperatures used in this study. The T_g was determined to be 218–221 °C from the linear intersection of the glassy and transition states in the E'-T curve and from the peak of the E''-T curve. The $\tan\delta$ curve increased to > 1 during the glass-to-rubber transition but showed no defined peak, so it was not used to report the T_g.

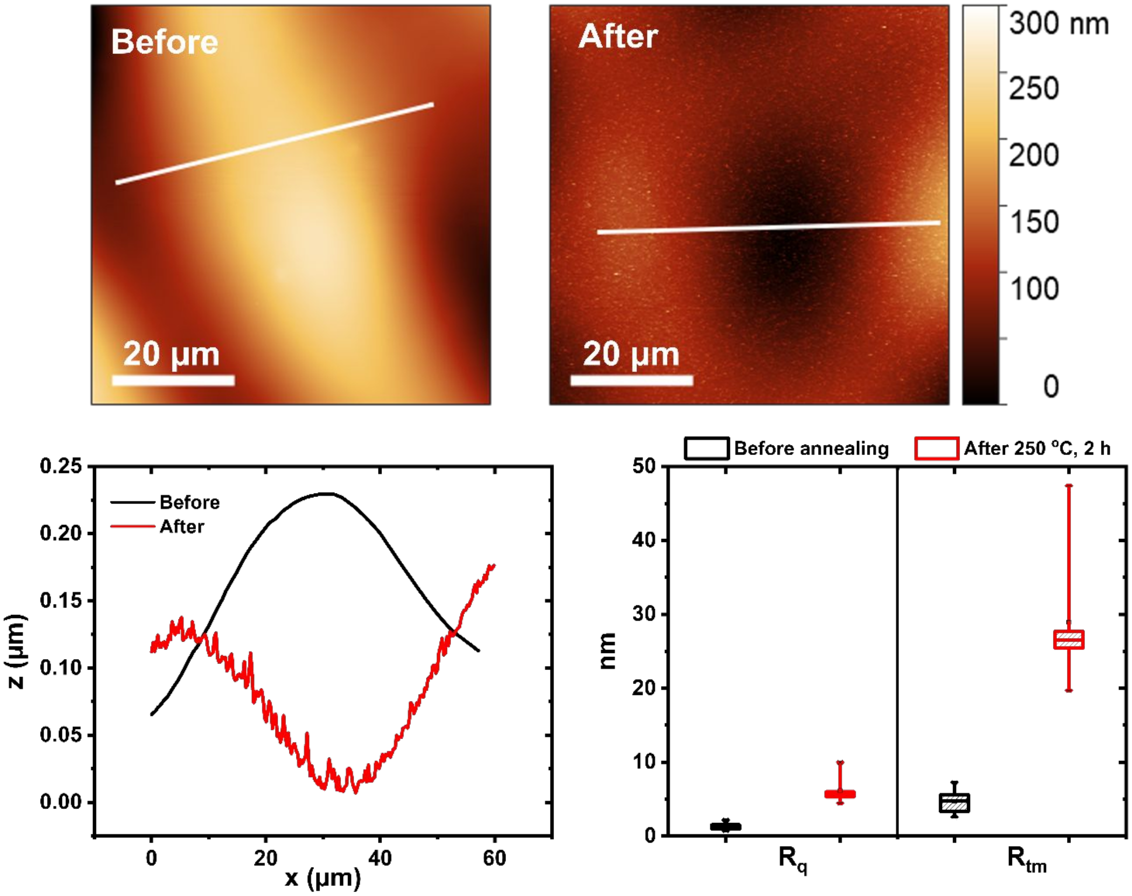

Figure S9. AFM images of PEI thin film spin-coated on a smooth glass slide (R_q < 1 nm) at 3000 rpm for 60 s using a solution of 50 mg/mL PEI in $CHCl_3$. The average peak-to-peak roughness height (R_{tm}) increased from 5 ± 2 nm before annealing to 29 ± 9 nm after annealing at 250 °C for 2 h, while the maximum waviness height decreased from 0.17 ± 0.02 μm to 0.10 ± 0.04 μm. The change in R_{tm} here indicates substantial roughening of the surface after annealing but with the absence of any 1D deformations like those seen in PEI/SLG annealed on Cu.

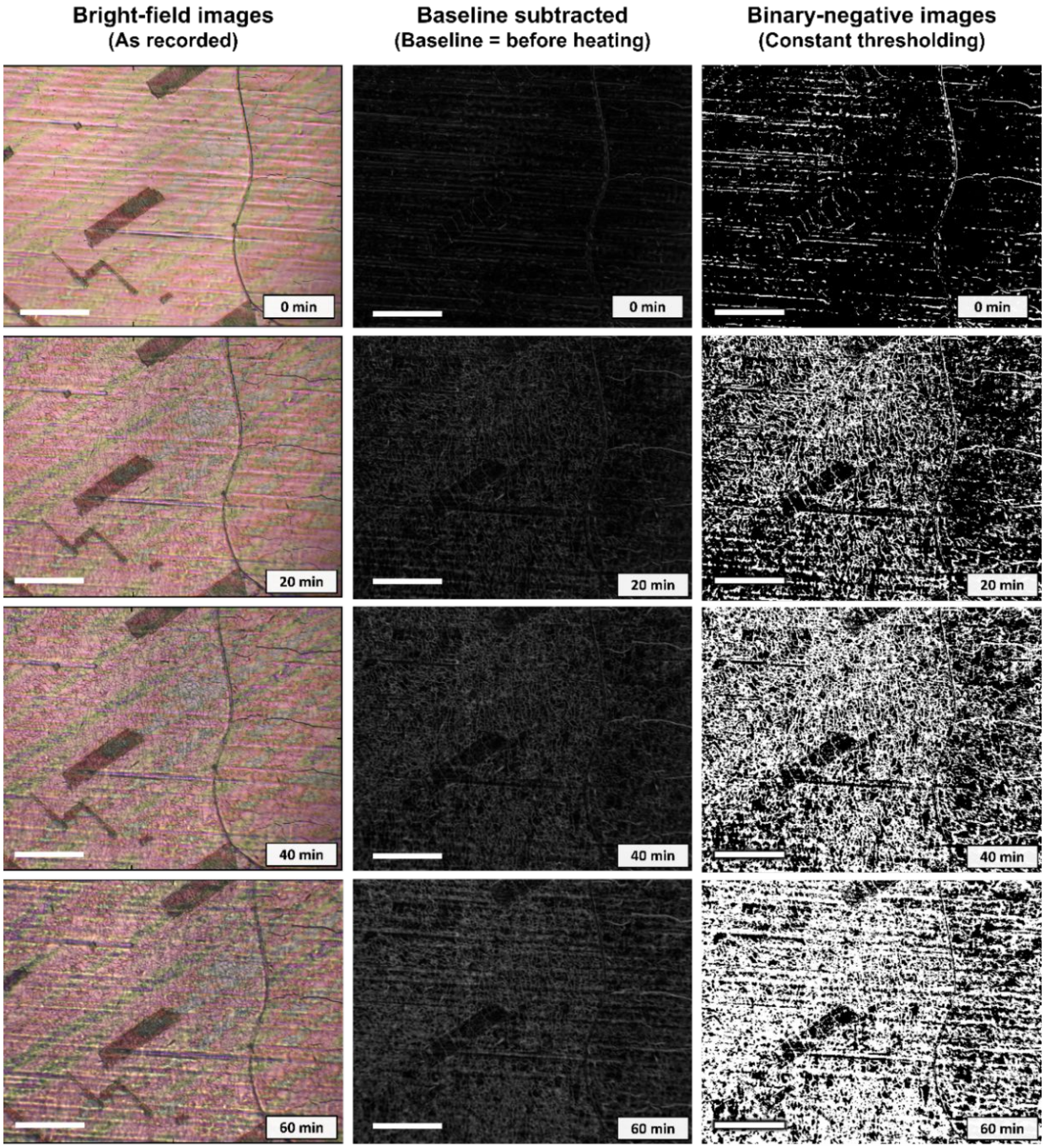

Figure S10. Image processing procedure for the quantification of areal deformation fraction over time. The difference between the bright field images (left) of a spot before thermal annealing and at each time interval during annealing is calculated to create "baseline subtracted" images (center) in grayscale. The baseline subtracted images are then converted to binary using a threshold of 30 (255-grayscale) to generate the binary-negative images (right), in which white pixels (1) and black pixels (0) correspond to deformed and undeformed areas of the surface, respectively. Scale bars, 200 μm.

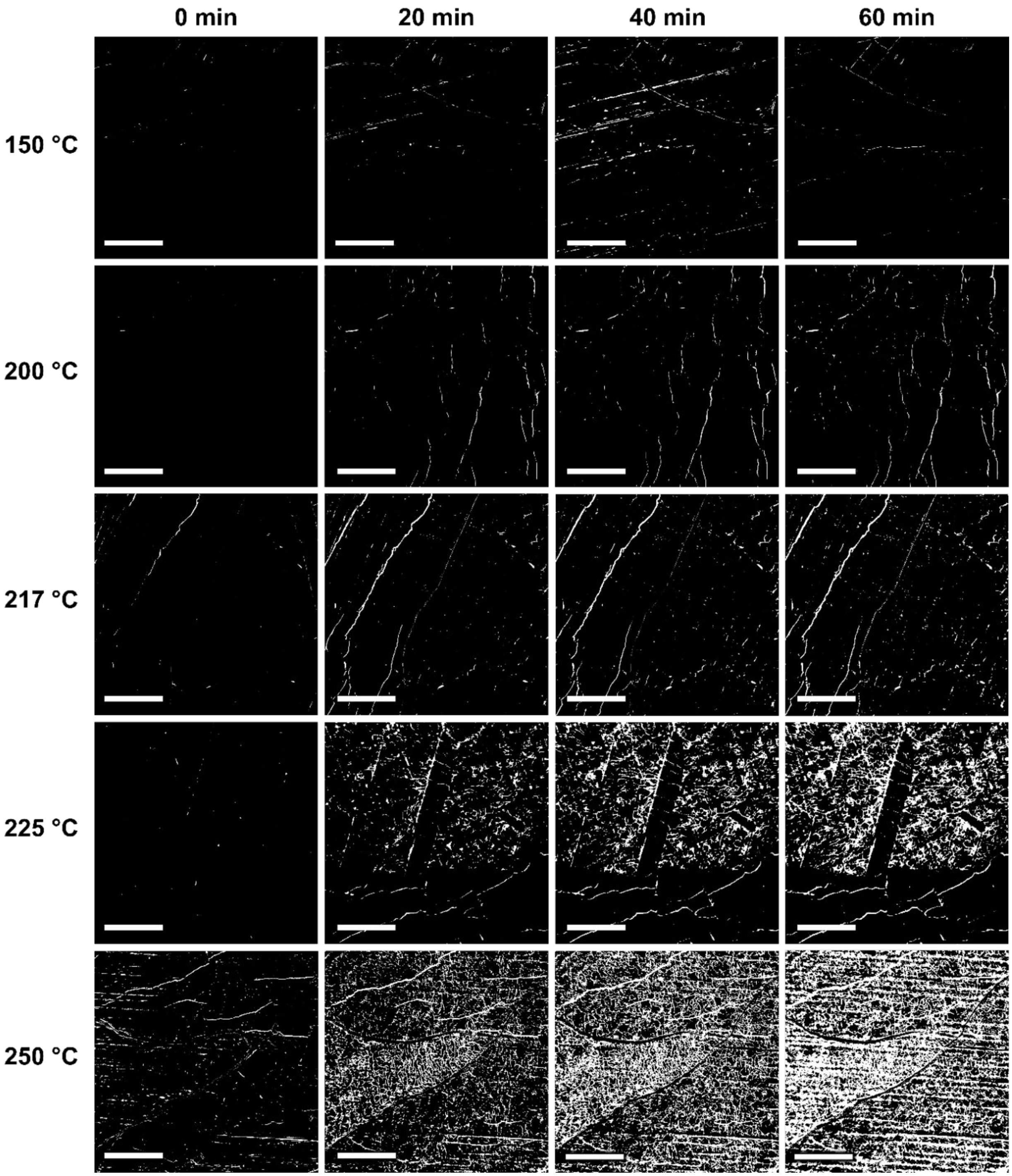

Figure S11. Processed binary-negative images highlighting the deformed areas in each image of Figure 5 (main text) and their appearance with time and temperature. At temperatures near and above the T_g of PEI (~217 °C),[2] thermal annealing-induced deformation begins to rapidly increase. Scale bars, 200 µm.

References

1. Schriver, M.; Regan, W.; Gannett, W. J.; Zaniewski, A. M.; Crommie, M. F.; Zettl, A., Graphene as a Long-Term Metal Oxidation Barrier: Worse Than Nothing. *ACS Nano* **2013,** *7* (7), 5763-5768.

2. SABIC, ULTEM™ RESIN 1010 Global Technical Datasheet. Online, 2023 [Date Accessed: May 14, 2024], https://www.sabic.com/en/products/specialties/ultem-resin-family-of-high-heat-solutions/ultem-resin.
